# Metabolism‐associated molecular classification of hepatocellular carcinoma

**DOI:** 10.1002/1878-0261.12639

**Published:** 2020-01-29

**Authors:** Chen Yang, Xiaowen Huang, Zhicheng Liu, Wenxin Qin, Cun Wang

**Affiliations:** ^1^ State Key Laboratory of Oncogenes and Related Genes Shanghai Cancer Institute Renji Hospital Shanghai Jiao Tong University School of Medicine China; ^2^ State Key Laboratory of Oncogenes and Related Genes Key Laboratory of Gastroenterology and Hepatology Ministry of Health Division of Gastroenterology and Hepatology Renji Hospital Shanghai Cancer Institute Shanghai Institute of Digestive Disease Shanghai Jiao Tong University School of Medicine China; ^3^ Hepatic Surgery Center Tongji Hospital Tongji Medical College Huazhong University of Science and Technology Wuhan China

**Keywords:** classification, hepatocellular carcinoma, immune signatures, metabolic genes

## Abstract

Hepatocellular carcinoma (HCC) is a disease with unique management complexity because it displays high heterogeneity of molecular phenotypes. We herein aimed to characterize the molecular features of HCC by the development of a classification system that was based on the gene expression profile of metabolic genes. Integrative analysis was performed with a metadata set featuring 371 and 231 HCC human samples from the Cancer Genome Atlas and the International Cancer Genome Consortium, respectively. All samples were linked with clinical information. RNA sequencing data of 2752 previously characterized metabolism‐related genes were used for non‐negative matrix factorization clustering, and three subclasses of HCC (C1, C2, and C3) were identified. We then analyzed the metadata set for metabolic signatures, prognostic value, transcriptome features, immune infiltration, clinical characteristics, and drug sensitivity of subclasses, and compared the resulting subclasses with previously published classifications. Subclass C1 displayed high metabolic activity, low α‐fetoprotein (AFP) expression, and good prognosis. Subclass C2 was associated with low metabolic activities and displayed high expression of immune checkpoint genes, demonstrating drug sensitivity toward cytotoxic T‐lymphocyte‐associated protein‐4 inhibitors and the receptor tyrosine kinase inhibitor cabozantinib. Subclass C3 displayed intermediate metabolic activity, high AFP expression level, and bad prognosis. Finally, a 90‐gene classifier was generated to enable HCC classification. This study establishes a new HCC classification based on the gene expression profiles of metabolic genes, thereby furthering the understanding of the genetic diversity of human HCC.

## Graphical abstract

Based on the gene expression profile of metabolic genes, patients with hepatocellular carcinoma (HCC) were divided into three subclasses: C1, C2, and C3. Each subclass had a different prognosis, clinicopathological characteristics, molecular characteristics, and potential therapies. Besides, this classification was associated with previously reported HCC molecular subclasses, including Boyault's classification, Chiang's classification, Hoshida's classification, Désert's classification, and the Cancer Genome Atlas's classification.

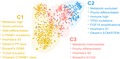

**Abbreviations**
AFP: α‐fetoprotein
CTLA‐4: cytotoxic T‐lymphocyte‐associated protein‐4
CTNNB1: cadherin‐associated protein beta 1
DEG: differentially expressed gene
ECM: extracellular matrix
GDSC: Genomics of Drug Sensitivity in Cancer
GEO: Gene Expression Omnibus
GSVA: gene set variation analysis
HBV: hepatitis B virus
HCC: hepatocellular carcinoma
HCV: hepatitis C virus
ICGC: the International Cancer Genome Consortium
MAD: median absolute deviation
MCP‐counter: microenvironment cell population‐counter
MST: median survival time
NMF: non‐negative matrix factorization
NTP: nearest template prediction
OS: overall survival
PD‐1: programmed cell death protein‐1
PP: periportal
PV: perivenous
TCGA: the Cancer Genome Atlas
Tem: effective memory T cell
Th1: T helper cell 1
Th17: T helper cell 17
Th2: T helper cell 2
Treg: regulatory T cells

## 1 Introduction

Hepatocellular carcinoma (HCC) is one of the most prevalent malignancies worldwide and the second leading cause of cancer‐related deaths. On the basis of annual projections, more than 1 million patients will die from HCC in 2030 according to the World Health Organization estimation (Karb and Sclair, 2019). Despite the current new treatments and diagnostic methods for HCC, its prognosis is still dismal (Hoshida *et al.*, 2014). It is critical to unravel the underlying molecular mechanisms of HCC diversity to develop targeted therapies (de Bono and Ashworth, 2010). In recent years, genome‐wide analyses of mRNA expression profiles have been devoted to this purpose (Boyault *et al.*, 2007; Chiang *et al.*, 2008; Hoshida *et al.*, 2009; Lee *et al.*, 2004). Although clinical samples were stratified in each transcriptome study, the correlations between the molecular and clinicopathological features have not been elucidated thoroughly.

Gene expression patterns of HCC are generally classified into two subclasses (Hoshida *et al.*, 2010; Llovet *et al.*, 2015; Zucman‐Rossi *et al.*, 2015), proliferative HCCs and nonproliferative HCCs, each representing 50% of tumors. Proliferative HCCs can exhibit specific characteristics by activating TGF‐β, MET, AKT, and/or IGF2 pathway. In comparison, nonproliferative HCCs are usually well‐differentiated, less aggressive, and present a lower serum α‐fetoprotein (AFP) levels, TP53 mutations, and better prognosis (Boyault *et al.*, 2007; Hoshida *et al.*, 2009; Lee *et al.*, 2004; Makowska *et al.*, 2016), and they tend to preserve the zonation program of distributing metabolic functions along the portocentral axis in normal liver (Ng *et al.*, 2017). The metabolic distinctions between proliferative HCCs and nonproliferative HCCs suggest the possibility to classify HCCs from the metabolic perspective to identify a certain subclass performing metabolic functions as normal livers with good outcome. Hence, 2752 metabolic genes encoded all human metabolic enzymes and small molecule transporters were obtained after literature screening in this study for unsupervised clustering.

A proteogenomic characterization published recently classified hepatitis B virus‐related HCC patients into three subgroups, namely metabolism subgroup (S‐Mb), microenvironment dysregulated subgroup (S‐Me), and proliferation subgroup. S‐Mb enriches in proteins involving cancer metabolism and has the best prognosis. S‐Me enriches in proteins involving immunity and inflammation and has worse prognosis compared with S‐Mb (Gao *et al.*, 2019). This research suggested the possibility to classify HCC from the metabolic prospective. It appears that human cancer mutations and cancer genes constantly affect metabolism processes including aerobic glycolysis, glutaminolysis, and one‐carbon metabolism which produces amino acids, nucleotides, fatty acids, and other substances for cell growth and proliferation (Fiehn *et al.*, 2016). Cancer is thought of as a metabolic disease due to metabolic disorder (Boroughs and DeBerardinis, 2015). In this study, two HCC cohorts were merged into a metadata set of 602 patients for clustering based on metabolic genes. Additional processed microarray data of 221 HCC samples were used for external validation. The unsupervised transcriptome analysis identified 3 subgroups of HCC, namely C1, C2, and C3. We evaluated the prognosis value, transcriptome features, correlations with metabolic signatures, immune infiltration, clinical characteristics, and drug sensitivity of the HCC subclasses, and compared them with previous classifications. Finally, a 90‐gene classifier was generated to determine the HCC classification.

## 2 Materials and methods

### 2.1 Patients and samples

Multiple data repositories, including the International Cancer Genome Consortium (ICGC, http://www.icgc.org), the Cancer Genome Atlas (TCGA, http://cancergenome.nih.gov/), Gene Expression Omnibus (GEO, http://www.ncbi.nlm.nih.gov/geo/), and Genomics of Drug Sensitivity in Cancer (GDSC, https://www.cancerrxgene.org/), were searched for available data for HCC. Datasets without enough samples (< 200) or clinical information were excluded. RNA sequencing data (raw counts) of 371 and 231 HCC human samples with available clinical information were retrieved from TCGA‐LIHC cohort and LIRI‐JP cohort, respectively, and raw counts were transformed into transcripts per kilobase million values for subsequent analysis. Next, two RNA‐seq datasets were merged into one metadata set, and the *combat* function in the sva r package (R Core Team, R Foundationfor Statistical Computing, Vienna, Austria) was applied to remove the batch effects. Figure S1 showed the principal component analysis before and after batch effect correction. Additional processed microarray data of 221 HCC samples from http://www.ncbi.nlm.nih.gov/geo/query/acc.cgi?acc=GSE14520 (based on GPL3921 platform) were used for external validation. In total, 823 HCC patients were enrolled in this study, and the patients' clinical characteristics are shown in Table 1. Gene somatic mutation data (MAF files) of LIHC and LIRI‐JP cohorts were achieved from TCGA and ICGC databases, respectively. Besides, copy number data of GISTIC2 for LIHC cohort were accessed from the GDAC FireBrowse (http://firebrowse.org/), and predicted neoantigens of LIHC cohort were achieved from previous analysis of the TCGA dataset (Rooney *et al.*, 2015). To investigate drug sensitivity, 16 hepatocarcinoma cell lines and 12 renal cell carcinoma cell lines with both gene expression data and drug sensitivity data (IC50 values) were also included in the analysis (Yang *et al.*, 2013).

**Table 1:** Clinical characteristics of TCGA, ICGC, and GEO sets. BCLC, Barcelona Clinic Liver Cancer; N/A, not available.

Variable	TCGA set	ICGC set	GEO set
	(*n* = 371)	(*n* = 231)	(*n* = 221)
Age			
≤ 55 years	125	25	152
> 55 years	245	206	69
Gender			
Female	121	61	30
Male	250	170	191
Viral infection			
HBV	95	N/A	221
HCV	49	N/A	0
HBV/HCV	7	N/A	0
No infection	103	N/A	0
Alcohol consumption			
Yes	115	N/A	N/A
No	103	N/A	N/A
Child‐Pugh score			
A	216	N/A	N/A
B/C	22	N/A	N/A
Tumor size			
> 5 cm	N/A	N/A	80
≤ 5 cm	N/A	N/A	140
Histologic grade			
G1	55	36	N/A
G2	177	105	N/A
G3	122	71	N/A
G4	12	19	N/A
TNM stage			
I/II	257	N/A	170
III/IV	90	N/A	49
BCLC stage			
0/A	N/A	N/A	168
B/C	N/A	N/A	51
AFP level			
Low	212	N/A	118
High	66	N/A	100
Vascular invasion			
None	206	N/A	N/A
Micro	93	N/A	N/A
Macro	16	N/A	N/A
Family history			
No	208	143	N/A
Yes	112	73	N/A

### 2.2 Identification of HCC subclasses

A previously published list of 2752 metabolism‐relevant genes encoding all known human metabolic enzymes and transporters was achieved for subsequent non‐negative matrix factorization (NMF) clustering (Possemato *et al.*, 2011). Before performing NMF, a filtering procedure was conducted. First, candidate genes of low median absolute deviation (MAD) value (MAD ≤ 0.5) across all the HCC patients were excluded. Then, Cox regression assessing the associations of all the candidate genes with overall survival (OS) was conducted using r package ‘survival’. Eventually, genes with high variable (MAD > 0.5) and significant prognostic value (*P* < 0.05) were used for sample clustering. Subsequently, unsupervised NMF clustering methods were performed using nmf r package on the metadata set (Gaujoux and Seoighe, 2010), and this method was also applied to http://www.ncbi.nlm.nih.gov/geo/query/acc.cgi?acc=GSE14520 by using the same candidate genes. The values of *k* where the magnitude of the cophenetic correlation coefficient began to fall were chosen as the optimal number of clusters (Brunet *et al.*, 2004). Class mapping (SubMap) analysis (Gene Pattern), a method to evaluate similarity of molecular classes between independent patient cohorts based on their expression profiles, was then used to determine whether the subclasses identified in the two above datasets were correlated. T‐distributed stochastic neighbor embedding (t‐SNE)‐based approach was then used to validate the subtype assignments using the mRNA expression data of above metabolic genes.

### 2.3 Gene set variation analysis

Gene set variation analysis (GSVA) is a nonparametric and unsupervised gene set enrichment method that can estimate the score of certain pathway or signature based on transcriptomic data (Hanzelmann *et al.*, 2013). The 115 metabolism‐relevant gene signatures and seven HCC progression‐relevant signatures were achieved from previously published studies (Desert *et al.*, 2017; Rosario *et al.*, 2018), and by using gsva r package, each sample received 120 scores corresponding to 115 metabolism signatures and seven progression‐relevant signatures. Subsequently, differential analysis was conducted based on the 113 metabolism scores using limma package in r software, and the signatures with an absolute log2 fold change (FC) > 0.2 (adjusted *P* < 0.05) were defined as differentially expressed signatures.

### 2.4 Estimation of immune infiltration

Microenvironment cell population‐counter (MCP‐counter), a methodology based on gene expression profile data, was used to evaluate absolute abundance of eight immune and two nonimmune stromal cell populations (immune cell populations: T cells, CD8 + T cells, natural killer cells, cytotoxic lymphocytes, B‐cell lineage, monocytic lineage cells, myeloid dendritic cells, and neutrophils; stromal cell populations: endothelial cells and fibroblasts) (Becht *et al.*, 2016). Besides, another approach for the estimation of immune infiltration used in this study was single‐sample GSEA (ssGSEA), which computed an enrichment score representing the degree to which genes in a particular gene set were coordinately up‐ or downregulated within a single sample (Barbie *et al.*, 2009). Using the gsva r package, additional 6 immune cell populations, including regulatory T cells (Treg), T helper cell 1 (Th1), T helper cell 2 (Th2), T helper cell 17 (Th17), central memory T cell, and effective memory T cell (Tem) were estimated. In addition, immune scores and stromal scores were calculated by applying the ESTIMATE algorithm, which can reflect the enrichment of stromal and immune cell gene signatures (Yoshihara *et al.*, 2013).

### 2.5 Characterization of HCC subclasses

The differentially expressed genes (DEGs) among HCC subclasses were identified using limma package in r on normalized count data. The genes with an absolute log2 FC > 1 (adjusted *P* < 0.01) were defined as DEGs. The gene set files of ‘c2.cp.kegg.v6.2.symbols’ and ‘h.all.v6.2.symbols’, downloaded from the Molecular Signatures Database, were employed for the functional and pathway enrichment analysis using the clusterprofiler r package, and the significance threshold was set at an adjusted *P* < 0.05. Prediction of previously published HCC molecular classifications was also performed using nearest template prediction (NTP) analyses (Gene Pattern modules), and the prediction results were then compared with our classification.

### 2.6 Generation of the classifier and performance validation

The statistically significant differential genes were defined as adjusted *P* < 0.01 and absolute log2 FC > 2. Only genes with significant differences in expression in all three possible comparisons were considered subclass‐specific genes. The top 30 genes with the largest log2FC value (only genes with log2 FC > 0 were chosen) in each subclass were further selected for the development of the prediction model, and thus, 90‐gene classifier was generated. Then, the subclass prediction was repeated with the 90‐gene signature on http://www.ncbi.nlm.nih.gov/geo/query/acc.cgi?acc=GSE14520 using NTP algorithm, and the result was compared with previous classification based on NMF algorithm.

### 2.7 Prediction of the benefit of each subclass from immunotherapy and targeted therapy

The available data from melanoma patients treated with immunotherapies were used to indirectly predict the immunotherapy's efficacy of our subclasses by measuring similarity of gene expression profiles between our subclasses and melanoma patients based on SubMap analysis (Gene Pattern) (Roh *et al.*, 2017). Besides, the drug sensitivity of two HCC‐targeted drugs, sorafenib (for first‐line treatment) and cabozantinib (for second‐line treatment), was also investigated using SubMap analysis on data derived from GDSC. Specifically, cell lines were ranked from low to high according to IC50 value, and cell lines in the top one‐third were defined as drug sensitivity, while cell lines in the last one‐third were drug resistance. Notably, considering all hepatocarcinoma cell lines have the same high IC50 value of sorafenib, we chose renal cell carcinoma cell lines for the prediction of sorafenib sensitivity.

### 2.8 Statistical analysis

All the computational and statistical analyses were performed using R programming (https://www.r-project.org/). Unpaired Student's *t*‐test was used to compare two groups with normally distributed variables, while Mann–Whitney *U*‐test was used to compare two groups with non‐normally distributed variables. For comparisons of three groups, one‐way analysis and Kruskal–Wallis tests of variance were used as parametric and nonparametric methods, respectively. Contingency table variables were analyzed by chi‐square test or Fisher's exact tests. Survival analysis was carried out using Kaplan–Meier methods and compared by the log‐rank test. A univariate Cox proportional hazards regression model was used to estimate the hazard ratios for univariate analyses. A two‐tailed *P* value < 0.05 was statistically significant.

## 3 Results

### 3.1 NMF identifies three subclasses in HCC

**Figure 1:**
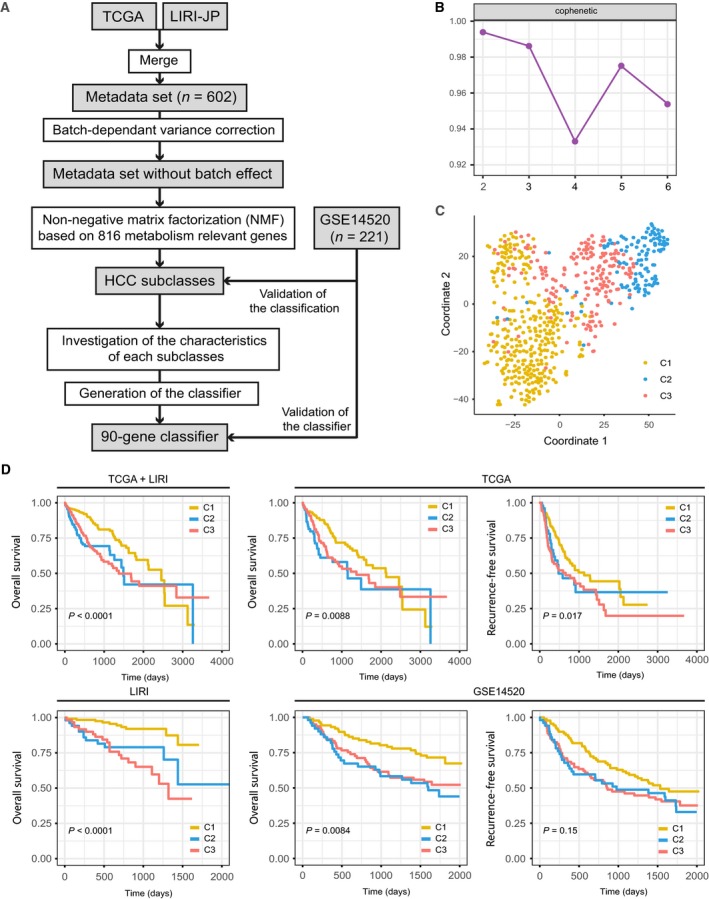
Identification of HCC subclasses using NMF consensus clustering in the metadata set. (A) Flow chart of the study. (B) NMF clustering using 816 metabolism‐associated genes. Cophenetic correlation coefficient for *k* = 2–5 is shown. (C) t‐SNE analysis supported the stratification into three HCC subclasses. (D). OS and RFS of three subclasses (C1, C2, and C3) in metadata set, independent TCGA or ICGC cohort, and http://www.ncbi.nlm.nih.gov/geo/query/acc.cgi?acc=GSE14520 cohort. The statistical significance of differences was determined by log‐rank test.

A flow chart was developed to systematically describe our study (Fig. 1A), and clinical characteristics of patients from different cohorts are shown in Table 1. Previously reported 2752 metabolism‐relevant genes were chosen as the basis of NMF analysis. After screening, a total of 816 candidate genes were identified (Table S1), and the metadata set comprising 602 HCC samples from TCGA and LIRI‐JP was clustered according to the expression profile of above‐mentioned 816 candidate genes using NMF consensus clustering. Cophenetic correlation coefficients were calculated to determine the optimal *k* value, and *k* = 3 was eventually chosen as optimal number of clusters after comprehensive consideration (Fig. 1B, three subclasses were designated C1, C2, and C3). When *k* = 3, the consensus matrix heatmap still keeps sharp and crisp boundaries, suggesting stable and robust clustering for the samples. To validate the subclasses' assignments, we also performed t‐SNE to decrease the dimension of features and found the subtype designations were largely concordant with two‐dimensional t‐SNE distribution patterns (Fig. 1C). Subsequently, we performed another independent analysis on a dataset with 221 HCC samples from GEO database (http://www.ncbi.nlm.nih.gov/geo/query/acc.cgi?acc=GSE14520), the results of which also revealed that there were three distinct molecular subclasses of HCC (Fig. S2A,B). A SubMap analysis was then conducted to determine whether the subclasses identified in the two above datasets were correlated, and the result showed that C1, C2, and C3 subclasses in metadata set were highly correlated with corresponding subclasses in http://www.ncbi.nlm.nih.gov/geo/query/acc.cgi?acc=GSE14520, suggesting there were three distinct molecular subclasses of HCC with different gene expression patterns (Fig. S3).

Using previously mentioned *k* = 3 classification, significant prognostic difference was observed in metadata set (log‐rank test *P* < 0.0001, Fig. 1D), with a longer median survival time (MST) for C1 (*n* = 307, MST = 2456 days, 95% CI: 2088–2824 days) than C2 (*n* = 117, MST = 1490 days, 95% CI: 1239–1741 days, *P* < 0.0001) and C3 (*n* = 178, MST = 1372 days, 95% CI: 914–1830 days, *P* < 0.0001). Independent TCGA and LIRI cohort also showed the same results (TCGA‐OS: log‐rank test *P* = 0.0088; TCGA‐recurrence‐free survival (RFS): log‐rank test *P* = 0.017; LIRI‐OS: log‐rank test *P* < 0.0001). Furthermore, prognostic difference was validated in http://www.ncbi.nlm.nih.gov/geo/query/acc.cgi?acc=GSE14520 cohort (221 patients with available survival information), and similar difference was also observed, with C1 showing a significantly longer OS time than that for C3 and C2 (*P* < 0.0001), while significant difference was not observed in RFS (log‐rank test *P* = 0.15).

### 3.2 Transcriptomes of the HCC subclasses

To better characterize the three HCC subclasses, differential analyses were performed. Gene expression differences were considered significant if the adjusted *P* value was < 0.01 and absolute log2 FC was > 2. Only genes with significant differences in expression in all three possible comparisons were considered subclass‐specific genes. Eventually, a total of 2830 subclass‐specific signature genes were identified, with 509 specific genes for C1, 2042 specific genes for C2, and 279 specific genes for C3 (Table S2). Next, Gene Oncology enrichment analysis of the signature genes was conducted using the clusterprofiler package, and significantly enriched biological processes are shown in Fig. S4 and Table S3. The specific genes of C1 and C2 showed enrichment of distinct biological processes. Numerous metabolism‐associated biological processes were significantly enriched for signature genes of C1, while abundant extracellular matrix (ECM)‐relevant processes were observed for signature genes of C2. For C3, it was enriched in some development‐relevant processes. Besides, GSEA was applied to identify pathways enriched in each subclass, the result of pathway analysis of subclass‐specific genes revealed that amino acid metabolism‐relevant pathways were significantly enriched for C1, ECM‐relevant pathways were enriched for C2, and other metabolism‐relevant pathways including hormone and proteoglycan metabolism were significantly enriched for C3 (Fig. S5, Table S4).

### 3.3 Correlation of the HCC subclasses with metabolism‐associated signatures

**Figure 2:**
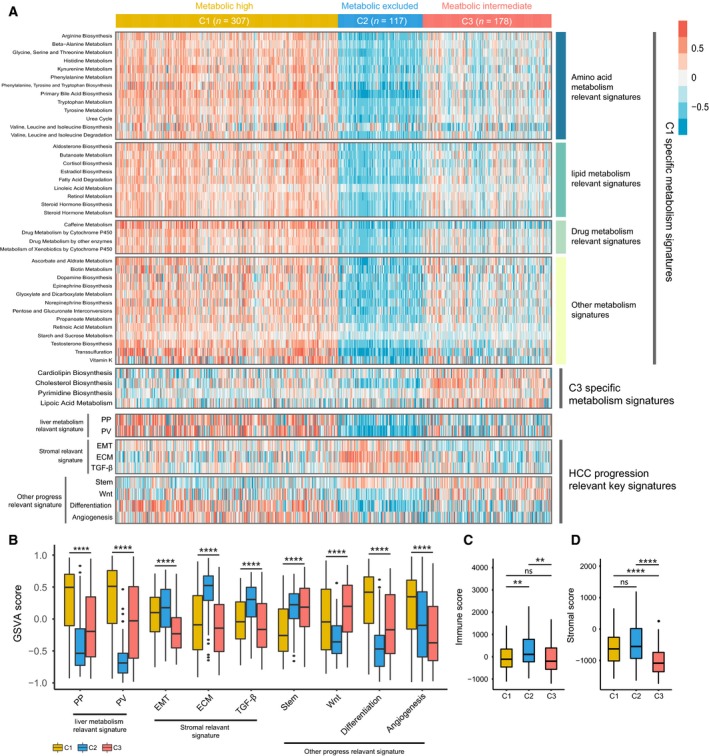
Association between metabolism and progression‐associated signatures and the HCC subclasses. (A) Heatmap of the specific metabolism‐associated signatures. (B) Boxplot of the signature score for HCC progression‐associated signatures distinguished by different subclasses. Boxplot of immune score (C) and stromal score (D) from ESTIMATE of three subclasses. For boxplots, the line within the boxes represents the median value, and the bottom and top of the boxes are the 25th and 75th percentiles (interquartile range), and the vertical line represents 1.5 times the interquartile range. The statistical difference was compared through the Kruskal–Wallis test, and the *P* values are labeled above each boxplot with asterisks (ns represents no significance, **P < 0.01, ****P < 0.0001).

Considering that the classification was based on metabolism‐relevant genes, we further explored whether distinct subclasses had different metabolic characteristics. First, 115 metabolism processes were quantified using gsva r package (Table S5). Then, differential analysis was conducted to find subclass‐specific metabolism signatures, which was defined as signature with higher GSVA score in the corresponding subclasses. Results showed that only C1 and C3 had specific metabolism signatures, and the numbers were 39 and 4, respectively, while C2 had no specific metabolism signatures according to the result of differential analysis. Notably, 13 of the 39 specific metabolism signatures in C1 were related to amino acid metabolism including urea cycle, which was similar to the metabolic patterns of previously reported periportal (PP)‐type HCC involving gene signatures of gluconeogenesis, amino acid catabolism, and urea cycle(Ng *et al.*, 2017) (Fig. 2A).

To further investigate the characteristics of subclasses, seven HCC‐associated key signatures were chosen and quantified using GSVA algorithm. C1 had significantly higher PP and perivenous (PV) signatures than C2 and C3, and C2 exhibited higher expression for stromal‐relevant signature, consistent with results from enrichment analysis. C1 had significantly lower score of stem‐relevant signature and higher score of differentiation‐associated signature than C2 and C3, which was corresponding to the clinical characteristics of C1. Besides, C1 and C3 both had significantly higher score of Wnt activation‐relevant signature than C2, which may be associated with their harboring high frequency of cadherin‐associated protein beta 1 (*CTNNB1*) mutations (Fig. 2B and Table S6). Then, ESTIMATE algorithm was used to calculate the immune and stromal score. Significant difference in immune score was observed among three groups, with higher immune score of C2 than C1 (*P* < 0.001) and C3 (*P* < 0.001; Fig. 2C). In addition, C3 exhibited lower stromal score than C1 (*P* < 0.00001) and C2 (*P* < 0.00001; Fig. 2D).

### 3.4 Correlation of the HCC subclasses with immune infiltration in the metadata set

**Figure 3:**
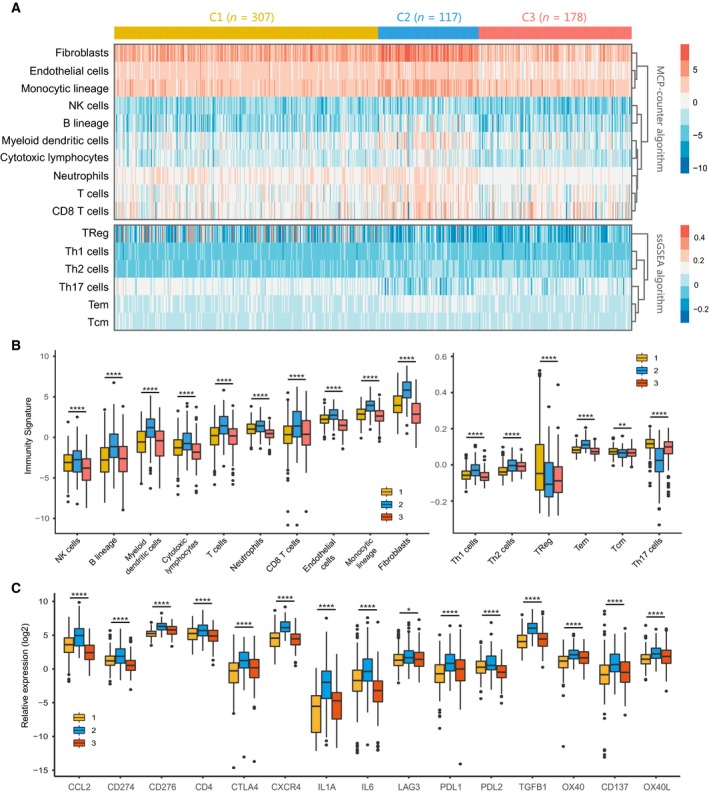
Immune characteristics of three subclasses in the metadata set. (A) Heatmap describing the abundance of immune and stromal cell populations in C1, C2, and C3. (B) Boxplot of the abundance of immune and stromal cell populations distinguished by different subclasses. (C) Expression level (normalized count) of 15 immune checkpoint genes in three HCC subclasses. The statistical difference was compared through the Kruskal–Wallis test, and the *P* values are labeled above each boxplot with asterisks (ns represents no significance, *P < 0.05, **P < 0.01, ****P < 0.0001).

With the significant difference in immune score identified among subclasses, immune infiltration was investigated to characterize their immunologic landscape. The abundance of 16 immune‐related cell types was calculated using MCP‐counter and ssGSEA algorithm and presented in a heatmap (Fig. 3A). Significant difference was observed between C2 and other two subclasses, with higher abundance of 11 immune cell populations (T cells, CD8 + T cells, NK cells, cytotoxic lymphocytes, B‐cell lineage, monocytic lineage cells, myeloid dendritic cells, neutrophils, Th1 cells, Th2 cells, and Tem cells) for C2 compared with C1 or C3. In addition, C2 also exhibited lower enrichment for Treg cells and Th17 cells. Notably, stromal cell populations (endothelial cells and fibroblasts) were significantly higher in C2, consistent with previous result of C2's enrichment for stromal‐relevant signatures (Fig. 3B). Detailed information is shown in Table S6.

We further investigated the association between subclasses and the expression of 15 potentially targetable immune checkpoint genes that were chosen based on current drug inhibitors in clinical trials or have been approved for specific cancer types, and the results indicated that C2 exhibited higher expression for 14 immune checkpoint genes (except for LAG3) than C1 and C3 (Fig. 3C).

### 3.5 Correlation of the HCC subclasses with clinical characteristics in the TCGA and GEO dataset

**Figure 4:**
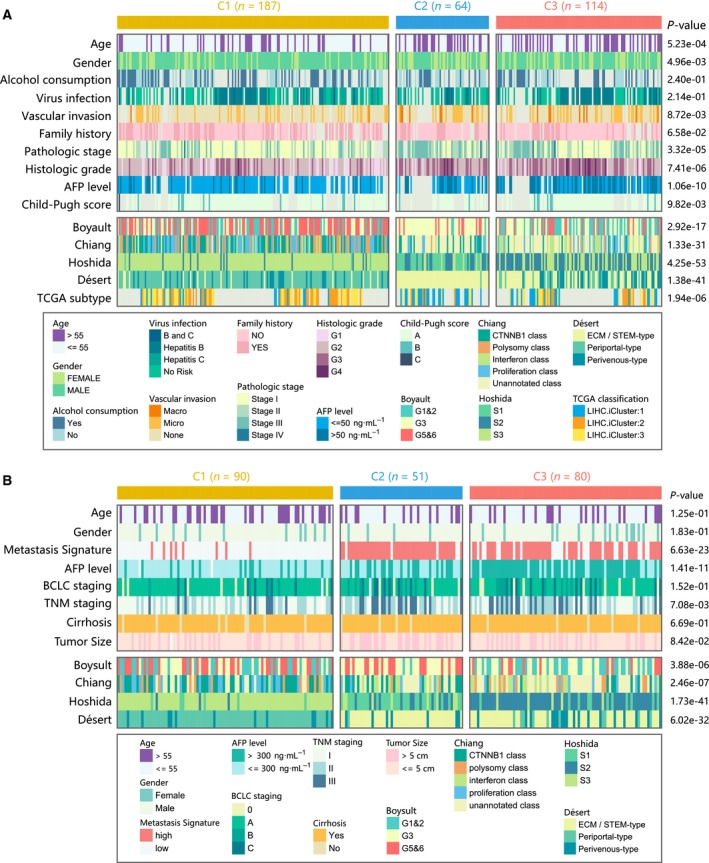
Clinical characteristics of HCC subclasses in the TCGA and http://www.ncbi.nlm.nih.gov/geo/query/acc.cgi?acc=GSE14520 cohort. (A) Correlation of our classification (C1, C2, and C3) with clinical characteristics and previous HCC subclasses in the TCGA cohort. (B) Correlation of our classification with clinical characteristics and previous HCC subclasses in http://www.ncbi.nlm.nih.gov/geo/query/acc.cgi?acc=GSE14520 cohort.

We then explored tumor‐related clinicopathological variables associated with our classification based on TCGA (Fig. 4A, Table S7) and GEO (Fig. 4B, Table S8) cohorts. The results of chi‐square test revealed several significant correlations between clinicopathological features and HCC subclasses in TCGA cohort. Lack of vascular invasion (*P* < 0.001), pathologic stage I/II (*P* < 0.001), histologic grade G1/G2 (*P* < 0.001), and low serum AFP level (*P* < 0.001) were associated with the C1 subclass, and presence of vascular invasion, advanced pathologic stage (III/IV), histologic grade (G3/G4), and high serum AFP level were associated with the C2 or C3 subclass. Similarly, in GEO cohort, C1 was correlated with low metastasis signature (*P* < 0.001), low serum AFP level (*P* < 0.001), and pathologic stage I/II (*P* = 0.001).

We also compared our classification with previously reported HCC molecular subclasses, including Boyault's classification (G1–G6), Chiang's classification (five classes), Hoshida's classification (S1, S2, and S3), Désert's classification (four classes), and TCGA classification (iCluster1, iCluster2, and iCluster3). In TCGA cohort, C1 subclass was significantly associated with Boyault's G5/G6 (*P* < 0.001), Chiang's proliferation (*P* < 0.001) and CTNNB1 class (*P* < 0.001), Hoshida's S3 (*P* < 0.001), Désert's PP‐type (*P* < 0.001), and TCGA iCluster2 (*P* < 0.001). C2 subclass was linked to Boyault's G3 (*P* < 0.001), Hoshida's S1 (*P* < 0.001), Désert's ECM/STEM‐type (*P* < 0.001), and TCGA iCluster1 (*P* < 0.001). C3 subclass was associated with Boyault's G1/G2 (*P* < 0.001) and Hoshida's S2 (*P* < 0.001). Similarly, in GEO cohort, C1 was linked to Boyault's G5/G6 (*P* = 0.023), Chiang's proliferation (*P* = 0.003) and CTNNB1 (*P* < 0.001) class, Hoshida's S3 (*P* < 0.001), and Désert's PP‐type (*P* < 0.001). C2 was linked to Hoshida's S1 (*P* < 0.001) and Désert's ECM/STEM‐type (*P* < 0.001). C3 was enriched in Boyault's G1/G2 (*P* = 0.004) and Hoshida's S2 (*P* < 0.001).

### 3.6 Correlation of the HCC subclasses with mutations, neoantigens, and copy number aberrations

**Figure 5:**
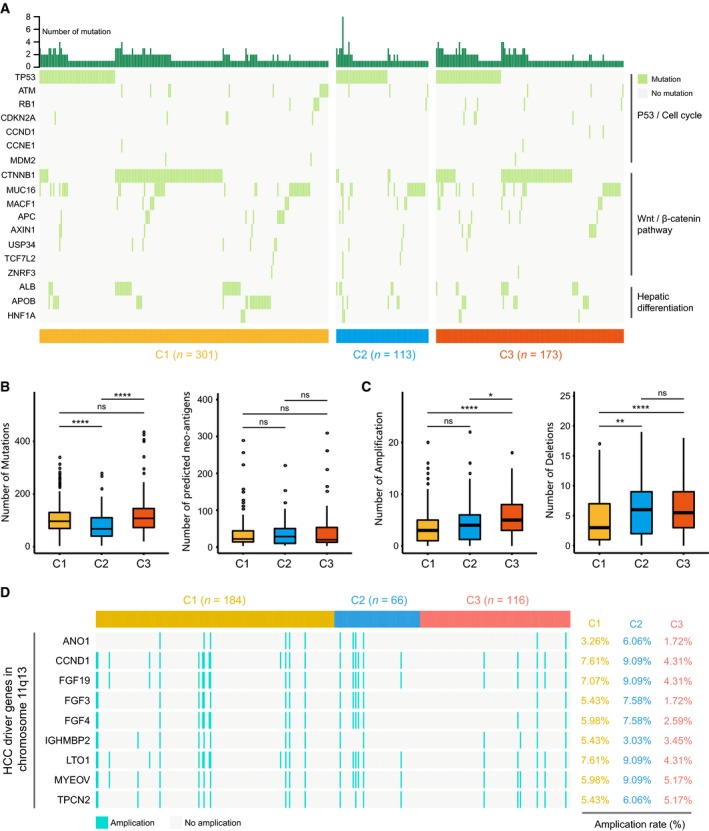
Association between HCC subclasses and mutations, neoantigens, and copy number aberrations. (A) Oncoprint of mutation status of genes in P53/cell cycle pathway, Wnt/β‐catenin pathway, and hepatic differentiation (see detailed statistical analysis in Table S9). The number of mutations, predicted neoantigens (B), and copy number aberrations (C) in HCC subclasses. Statistical difference was compared through Wilcoxon rank‐sum test (ns represents no significance, *P < 0.05, **P < 0.01, ****P < 0.0001). (D) Amplification rate of HCC driver genes on chromosome 11q13 in HCC subclasses.

The tumoral genomic landscape has been proven to be correlated with antitumor immunity. To investigate whether differences exist in the somatic mutation frequencies across HCC subclasses and observe different patterns of mutations among HCC clusters, somatic mutation data from TCGA and ICGC databases were analyzed. The genes with high mutation frequency or in critical pathways, including P53/cell cycle pathway, Wnt/beta‐catenin pathway, and hepatic differentiation, are visualized in Fig. 5A (detailed statistical analysis is shown in the Table S9). Results showed that C1 and C2 displayed distinct mutation characteristics. Specifically, C1 had significantly lower mutation frequency of TP53 (16%) than C2 (30%) and C3 (25%), while C2 had significantly lower mutation frequency of *CTNNB1* (3%) than C1 (26%) and C3 (35%). Notably, although C3 exhibited higher mutation frequency of *CTNNB1* than C1 and C2, other results of this study did not support characterizing C3 as a subclass of frequent *CTNNB1* mutations.

We then correlated the classification with the number of overall mutations and predicted neoantigens (Fig. 5B). And significant difference was observed in the number of mutations, with a smaller median number of mutations for C2 (*n* = 67) compared with C1 (*n* = 96, *P* < 0.00001) and C3 (*n* = 107, *P* < 0.00001), respectively. No statistical difference was identified for the number of neoantigens in pairwise comparison (Fig. 5B). In terms of somatic copy number aberrations, patients within C1 showed lower burden of both gains and losses than C3, with a median of three broad gains (range 0–20) and three broad losses (range 0–17) in C1 vs five broad gains (range 0–18, *P* < 0.00001) and 5.5 broad losses (range 0–18, *P* < 0.00001) in C3. There was no statistical difference in burden of gains between C1 (three broad gains, range 0–20) and C2 (four broad gains, range 0–22, *P* = 0.078); however, C2 (six broad losses, range 0–19) had higher burden of losses than C1 (three broad losses, range 0–17, *P* < 0.001; Fig. 5C).

Previous study indicates that HCC driver genes on chromosome 11q13 (eg, FGF19) have higher possibility of amplification (Schulze *et al.*, 2015; Sia *et al.*, 2017). These genes may exert critical function in the treatment of HCC. Therefore, we next investigated the correlation between the HCC classification and the amplification of driver genes on chromosome 11q13 (Fig. 5D). Although no significant difference in driver genes' amplification was observed between pairs of subclasses, C2 still showed a trend toward higher amplification rate (e.g., FGF19: 9.09%) than C1 (7.07%, *P* = 0.59) and C3 (4.31%, *P* = 0.21; detailed statistical analyses are shown in Table S10).

### 3.7 Ninety‐gene classifier and performance validation

**Figure 6:**
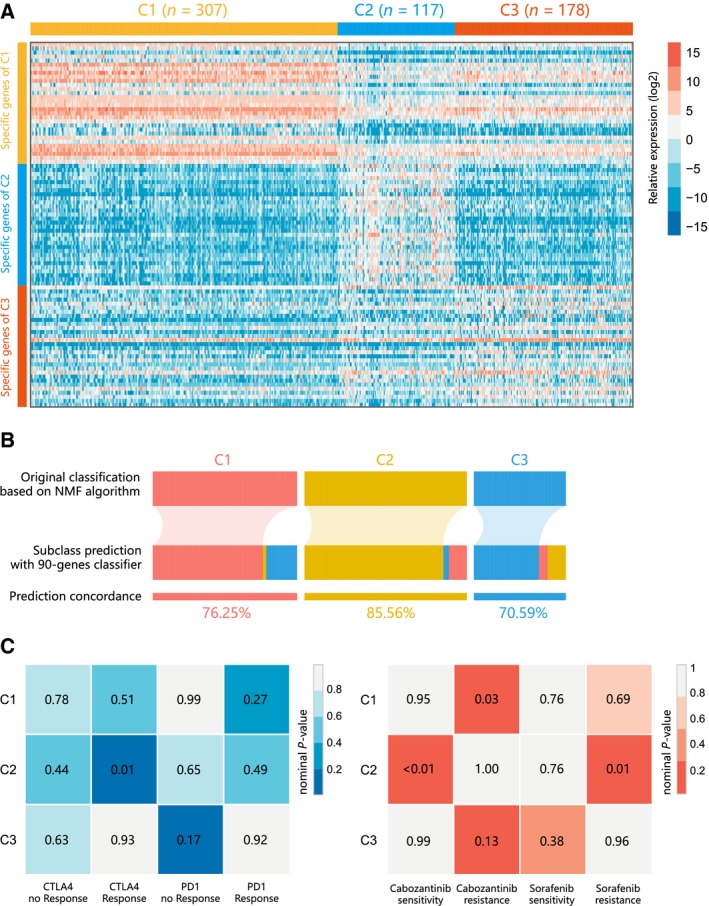
Identification of predictive classifier and putative targeted therapeutic and immunotherapeutic response. (A) Heatmap of the expression level of the 90‐gene classifier. (B) Concordance of HCC molecular subclass prediction between the 90‐gene classifier and original prediction based on NMF. (C) C2 may be more sensitive to the CTLA‐4 inhibitor (nominal *P* = 0.01) and cabozantinib (nominal *P* < 0.01) by SubMap analysis.

Differential analysis yielded 509 significant genes for the C1 subclass, 2042 for the C2 subclass, and 279 for the C3 subclass. To build a classifier for clinical use, it is necessary to select top informative subclass‐associated signature genes. After comprehensive consideration of accuracy and clinical application potential, top 30 genes with largest log2FC value (> 0) in each subclass were selected for the development of the subclasses' classifier. Thus, a 90‐gene classifier was generated and visualized in Fig. 6A and Table S11. Subsequently, the subclass prediction was repeated with the 90‐gene classifier in http://www.ncbi.nlm.nih.gov/geo/query/acc.cgi?acc=GSE14520 datasets (Fig. 6B). The concordance with the original prediction based on NMF was evaluated, and we observed the concordance of 76.35% in C1 subclass, 85.56% in C2 subclasses, and 70.59% in C3 subclasses. Results suggested that the 90‐gene signature can reproducibly determine the HCC classification.

### 3.8 Distinct sensitivity to immunotherapy and targeted therapies for HCC subclasses

Different immune infiltration patterns and expression levels of immune checkpoint genes among HCC subclasses indicated that the likelihood of responding to immunotherapy needed to be further investigated. Using subclass mapping, we compared the expression profiles of three HCC subclasses (C1, C2, and C3) with another published dataset containing 47 patients with melanoma that received programmed cell death protein‐1 (PD‐1) immune checkpoint inhibitor or cytotoxic T‐lymphocyte‐associated protein‐4 (CTLA‐4) immune checkpoint inhibitor (Fig. 6C). Significant correlation was observed when comparing the expression profile of C2 group with CTLA4‐response group (*P* = 0.01), indicating that patients within C2 group were more promising to respond to anti‐CTLA4 therapy.

Besides, we also explored the association between HCC subclasses and sensitivity toward targeted drugs (sorafenib and cabozantinib) using the same method (Fig. 6D). For cabozantinib, C2 exhibited significant association of cabozantinib‐sensitive group (*P* < 0.01), while C1 was significantly associated with cabozantinib‐resistant group (*P* = 0.03). For sorafenib, significant correlation was only observed between C2 group and sorafenib‐resistant group (*P* = 0.01).

## 4 Discussion

Although numerous HCC classifications based on gene expression have been proposed in recent years, a consensus in molecular taxonomy has not yet been established. To identify HCC subgroup associated with metabolic processes and good prognosis, HCC classification was established in this study based on 2752 metabolic genes screened from previous publications. Three subclasses of HCC (C1, C2, and C3) were identified. The metabolic signatures, prognosis value, transcriptome features, immune infiltration, clinical characteristics, and drug sensitivity of the subclasses were explored. Results showed that C1 displayed distinct metabolic signatures and was similar to the differentiated nonproliferative HCCs with low AFP and good prognosis. C2 was associated with immunity signatures and had high expressions of immune checkpoint genes, demonstrating drug sensitivity toward CTLA4 inhibitors and cabozantinib. This class was barely involved in metabolic signatures. C3 with higher level of AFP and worse prognosis demonstrated less enrichment in metabolic signatures than C1 but higher enrichment of metabolic signatures than C2. In general, this study explored the metabolic landscape of HCC and identified three clusters with active, intermediate, or exhausted metabolic activities, respectively.

Liver parenchymal cells are critical in the metabolic processes of HCC and present a gradient pattern along the portocentral axis. For example, gluconeogenesis, amino acid catabolism, and urea synthesis are performed in PP hepatocytes, while lipogenesis and glycolysis are increased in the PV hepatocytes (Ng *et al.*, 2017). A classification model of HCC provided by Désert et al revealed four subclasses, namely ‘ECM‐type’, ‘STEM‐type’, ‘PV‐type’, and ‘PP‐type’ (Desert *et al.*, 2017). The ECM type featured in signatures of ECM modeling, integrin signaling, and epithelial‐to‐mesenchymal transition, which is in accordance with the C2 subclass characterizing by stromal‐relevant signature and abundant gene signatures of ECM‐relevant processes in our study. The PP and PV types are two distinct subclasses of nonproliferative HCCs. The PP HCCs preserve the default metabolic program of normal liver and are well‐differentiated and enriched for signatures of PP hepatocytes including gluconeogenesis, amino acid catabolism, urea cycle, and HNF4A‐induced genes. Therefore, the PP program is the least aggressive in HCCs and is correlated with favorable survival and low recurrence. The PV subclass is highly enriched in lipid and bile salt metabolism signatures and activates the WNT signaling pathway, presenting high frequency for predicted CTNNB1 mutations.

C1 was chiefly involved in amino acid metabolism including urea cycle and lipid metabolism processes as well as differentiation signatures, indicating better prognosis compared with C2 and C3 involving in HCC progression signatures (EMT, ECM, TGF‐β/SMAD, Stem, Wnt/β‐catenin). C1 showed similar metabolism patterns of the PP and PV subclasses combined. The enrichment in metabolic signatures indicated that C1 patients may be beneficial from metabolic therapies. Metabolic therapies targeting certain metabolism processes provide alternatives for chemoresistant patients. For example, it has been reported that metformin can prevent liver carcinogenesis (Shankaraiah *et al.*, 2019) and treatment with metformin is associated with favorable prognosis in patients with HCC (Schulte *et al.*, 2019). Determining the responders of metabolic therapies has proven to be challenging (Rosario *et al.*, 2018). This study provided insights into predicting potential responders toward metabolic therapies. In addition, the lowest score of stem‐relevant signature and highest score of differentiation of C1 corresponded with its clinical characteristics of lack of vascular invasion, pathologic stage I/II, and histologic grade G1/G2. Further, it has been validated in previous study (Hoshida *et al.*, 2009) that subclass with elevated AFP indicates poor prognosis, which may be a possible explanation for the relationship between the lowest serum AFP level and favorable survival in C1.

C2 matched the G3 (Boyault *et al.*, 2007) subclass in terms of the highest mutation frequency in TP53. HCCs bearing TP53 mutations are often associated with a high level of chromosome instability and poor prognosis (Laurent‐Puig *et al.*, 2001). Nowadays, immunotherapy has gained widespread attention in cancer treatment. The safety and efficacy of several PD‐1 immune checkpoint inhibitors and CTLA‐4 inhibitors have been evaluated, and the outcomes are promising (Duffy *et al.*, 2017; El‐Khoueiry *et al.*, 2017; Killock, 2017). The highest infiltration of stromal cell identified in C2 corresponded with its enrichment in stromal‐relevant signatures. An HCC classification based on immune microenvironment reported favorable prognosis in subclass with abundant immune infiltration and poor prognosis in immune‐low subclass (Kurebayashi *et al.*, 2018), contradicting with the unfavorable prognosis of the immune‐high C2 in our study. The HCC microenvironment consisted of immune‐suppressive cells and high expression of immune checkpoint molecules. T‐cell exhaustion, tumor‐specific T‐cell dysfunction, and immune evasion by tumor cells are the results of the interaction between PD‐1 and PD‐L1 on tumor‐infiltrating lymphocytes and tumor cells, respectively. Additional inhibitory molecules will be expressed by the exhausted T cells depending on the severity of exhaustion, which may reverse by combined PD‐1/CTLA‐4 blockade (Wherry and Kurachi, 2015). High expression of PD‐L1 is often associated with high expression of PD‐1 on CD8 + T cells, indicating a poorer prognosis due to higher risk of cancer recurrence or metastasis and cancer‐related death (Dai *et al.*, 2017). Furthermore, an increased number of Treg can suppress immune response to tumor cells by inhibiting proliferation, activation, degranulation, and production of perforin and granzymes by CD8 + T cells (Chen *et al.*, 2003), leading to poor disease prognosis (Gao *et al.*, 2007). In addition, a systematic review reveals that Th17 cells can both increase tumor progression and mediate antitumor immune response, but generally, they are correlated with enhanced prognosis in cancers (Punt *et al.*, 2015). The poor prognosis in C2 may be attributed to the combined effects of low infiltration of Treg and Th17, high expression of immune checkpoint genes, presence of vascular invasion, advanced pathologic stage (III/IV), histologic grade (G3/G4), and high serum AFP level. On the other hand, the highest expression of 14 out of 15 immune checkpoint genes in C2 provided possibility of immunotherapy for C2 patients. Results indicated that C2 was promising toward anti‐CTLA4 therapy and cabozantinib but not sorafenib. Cabozantinib is a second‐line therapy and is available for clinical use during the 2‐year period from 2017 through 2018 (Kudo, 2018). The high expression of CTLA4 may account for C2's sensitivity toward anti‐CTLA4 therapy. The outcome of our study provides a novel insight into the combination therapy of anti‐CTLA4 therapy and cabozantinib, which requires further validation in large cohorts.

Particularly, it has been demonstrated that neoantigens load and overall mutations load may drive T‐cell responses (Diaz and Le, 2015; McGranahan *et al.*, 2016). Therefore, we made efforts to verify whether copy number aberrations (deletions and amplifications), number of mutations, and neoantigens are associated with immune infiltration in HCC. In this study, no association was detected between neoantigen load and the subclasses, and C2 was correlated with the least number of mutations. In terms of copy number aberrations, C2 patients showed lower burden of gains but higher burden of losses. Our data suggested that neither neoantigen load nor mutational load was correlated with T‐cell response, but copy number changes may have an effect on the immune response. The antitumor immunity in HCC may be driven by other mechanisms, such as the quality or clonality of neoantigens, expression of HCC‐associated antigens, and aneuploidy and mutations in specific oncogenic pathways (Charoentong *et al.*, 2017; McGranahan *et al.*, 2016; Sia *et al.*, 2017). Although no significance was detected for the amplification of the locus 11q13 in C2, C2 showed higher amplification rate of HCC driver genes compared with C1 and C3, indicating an association between immune infiltration and oncogenesis. Notably, patients with genomically amplified FGF19 can possibly gain from therapy targeting fibroblast growth factor receptor 4 (FGFR4). A FGFR4‐targeted drug, BLU‐554, has been evaluated in a Phase 1 clinical trial by Blueprint Medicines and the result showed great potential for controlling HCC progression (https://clinicaltrials.gov/ct2/show/NCT02508467). Further clinical trials are required to test the efficacy of combination of anti‐CTLA4 therapy and anti‐FGFR4 therapy for C2 patients.

According to the results, a larger proportion of C1 (26%) and C3 (35%) patients carried *CTNNB1* mutations compared with C2 (3%). C1 matched Hoshida's S3, Chiang's *CTNNB1* subclass, and Boyault's G5/G6 related to *CTNNB1* mutations that lead to Wnt pathway activation. The explanation for higher proportion of *CTNNB1* mutations in C3 compared with C1 should be referred to the result of t‐SNE analysis. C1 separated into two subpopulations, and some outliers of C3 mixed with C1. We speculated that C1 showing heterogeneity can be potentially subdivided into two distinct subtypes with different patterns of *CTNNB1* mutations, and either subtype may have a larger proportion of patients bearing *CTNNB1* mutations compared with C3. In addition, C3 and C1 showed similar molecular patterns. Higher AFP level and worse prognosis as well as lower abundance in metabolic signatures distinguished C3 from C1. C3 outliers mixed in the C1 population had great possibility in carrying *CTNNB1* mutations due to the genetic profiles of C1, contributing to larger proportion of *CTNNB1* mutations in C3. A more reasonable outcome will be attained if these C3 outliers are relabeled as C1.

The high frequency of *CTNNB1* mutations in C1 indicated that patients of this subclass may be beneficial from Wnt signaling pathway‐targeted inhibitors. The Wnt/β‐catenin signaling pathway may be the best characterized oncogenic pathway in HCC (Hoshida *et al.*, 2009). *CTNNB1*‐activating mutations are identified in ~ 11–41% of liver cancers (Guichard *et al.*, 2012). Wnt signaling activation mainly due to mutations in *CTNNB1*, a β‐catenin gene, has been recognized in a major subset of HCC patients (Delgado *et al.*, 2015). Previous studies have revealed that tumor‐intrinsic active β‐catenin signaling may lead to T‐cell exclusion, thus resistance toward anti‐PD‐L1 and anti‐CTLA4 (Spranger *et al.*, 2015), which is consistent with the insensitivity toward immune blockade in C1 patients of our study. Clinical testing of the sensitivity toward *CTNNB1*‐targeted inhibitors may be promising for C1 patients. Because of the worse prognosis and nondistinctive character of C3, there may be less treatment options for C3 patients.

In general, the classification we established validated the findings of previously established HCC subclasses, but at the same time preserved its own features. Specifically, this classification perfectly matched the 3 subclasses from Hoshida (S1, S2, and S3). C1 matched Hoshida's S3 and presented the characteristics of well‐differentiated, nonproliferative HCC. C2 and Hoshida's S1 coincided in poor differentiation with poor prognosis and high immune infiltration. C3 corresponded with Hoshida's S2 in high AFP level and poor prognosis. Thus, this work is a new proof of the existence of Hoshida's subclasses in TCGA‐LIHC cohort and LIRI‐JP cohort combined. In addition, this study not only validated the clinical significance of Hoshida's classification, but also unveiled unexploited features of Hoshida's classification. By classifying HCC into three clusters with active, intermediate, or exhausted metabolic activities, this study provided new insights into the heterogeneity of HCC from the metabolic landscape and proposed possible clinical treatment options for HCC subtypes. We also highlighted for the first time that C2, corresponding to Hoshida’s S1, was more likely to be responders of immune checkpoint inhibitors.

## 5 Conclusion

In conclusion, this study classified HCCs from the metabolic perspective and proposed three subclasses with active, intermediate, or exhausted metabolic activities, respectively. C1 was intensively correlated with metabolic processes with good prognosis, matching characteristics of the established nonproliferative HCCs. C2 exhibited high immune infiltration and sensitivity toward immune blockade as well as chemotherapy. C3 with higher level of AFP and worse prognosis was less active in metabolism compared with C1 but more active than C2. With the high predictive value of the 90‐gene classifier, our classification may help to predict the prognosis of HCC patients and prospective therapies.

## Conflict of interest

The authors declare no conflict of interest.

## Author contributions

CY and ZL were responsible for the analysis, interpretation of data, and graphing. XWH drafted the manuscript. All authors read and approved the final manuscript. WQ and CW supervised the whole analysis and provided guidance and instructions.

## Consent for publication

Consent to publish has been obtained from all authors.

6

## Supporting information


**Fig. S1.** The principal component analysis (PCA) before and after batch effect correction.

**Fig. S2.** (A) Consensus matrix of NMF clustering for *k* = 2–6 in TCGA cohort. (B) Consensus matrix of NMF clustering for *k* = 2–6 and cophenetic correlation coefficient under corresponding *k* values in GEO cohort.

**Fig. S3.** Submaps matrix shows significant correlation of HCC classification from independent datasets.

**Fig. S4.** Gene Ontology (GO) enrichment analysis of subclass‐specific genes. The x axis indicates the number of genes within each GO term. Detailed information in Table S4.

**Fig. S5.** Results of gene set enrichment analysis of subclass‐specific genes are shown. Detailed information in Table S5.

**Table S1.** The 816 metabolism associated genes used for classification.
**Table S2.** The result of differential expression analysis.
**Table S3.** Functional enrichment analyses of subclass specific genes.
**Table S4.** Pathway enrichment analysis of three HCC subclasses.
**Table S5.** 113 metabolism associated signatures.
**Table S6.** The result of pairwise comparison.
**Table S7.** Clinical Characteristics of patients with distinct classification in TCGA cohort.
**Table S8.** Clinical Characteristics of patients with distinct classification in GEO cohort.
**Table S9.** Mutation characteristics in distinct HCC classification.
**Table S10.** Distribution of amplifications in HCC driver genes on chromosome 11q13 among three subclasses.
**Table S11.** The 90‐gene classification signature.

## Data Availability

All data in our study are available upon request.
