# Supplementary figures and images for: Metabolism‐associated molecular classification of hepatocellular carcinoma
Source: Mol Oncol. 2020 Jan 29;14(4):896–913. doi: 10.1002/1878-0261.12639 (PMC7138397; doi:10.1002/1878-0261.12639)

Before removing batch effect

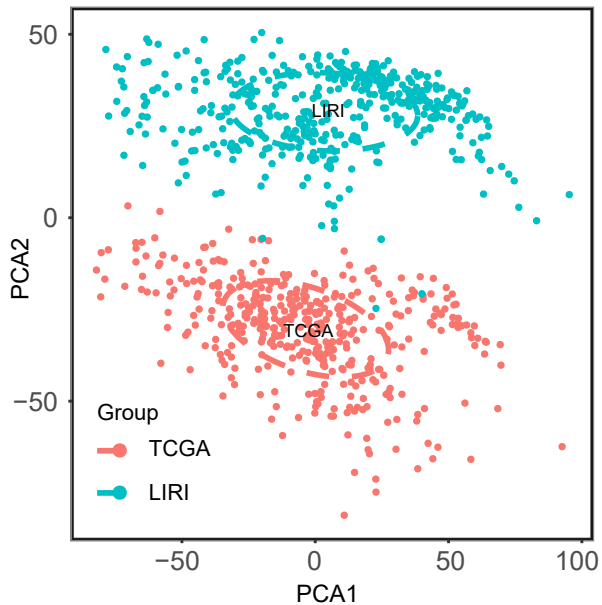

After removing batch effect

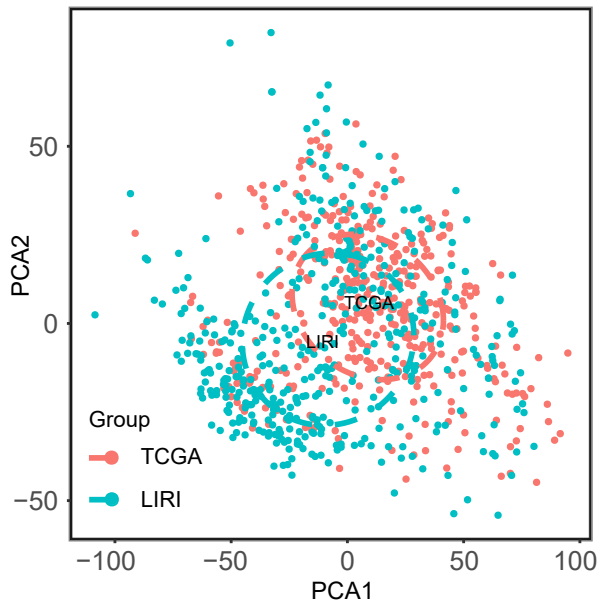

Supplement: Supplementary file 1 — Fig. S1. The principal component analysis (PCA) before and after batch effect correction. [file MOL2-14-896-s001.pdf]

**A**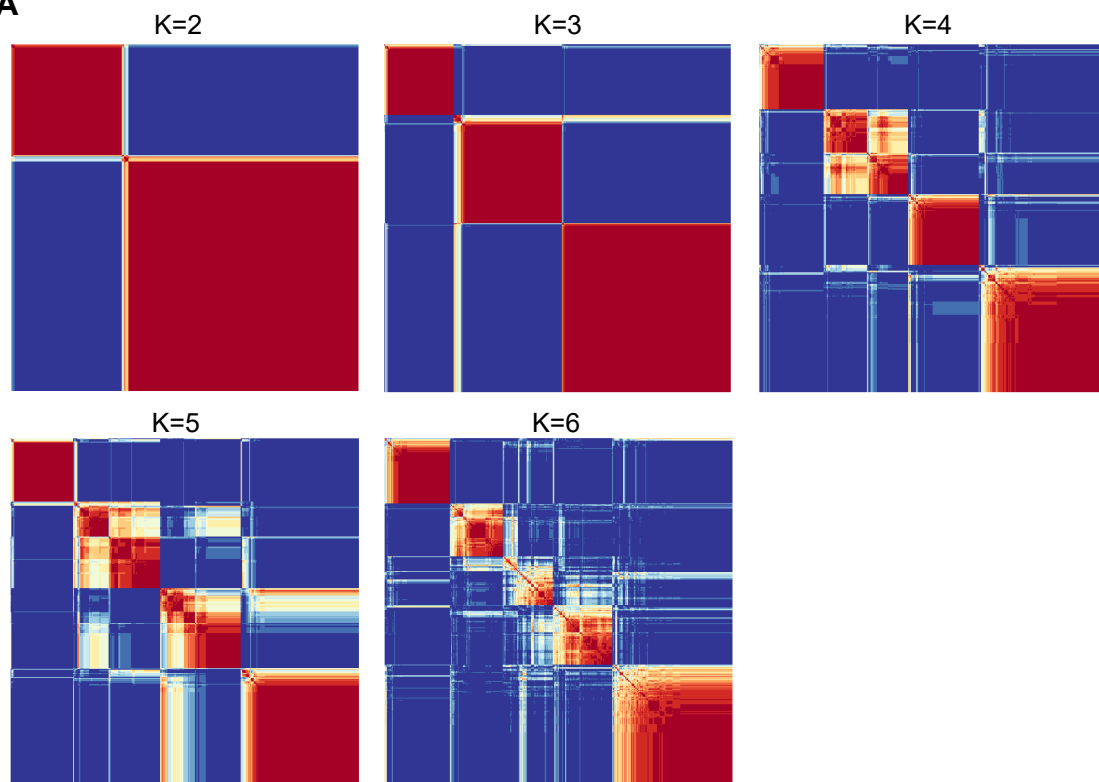**B**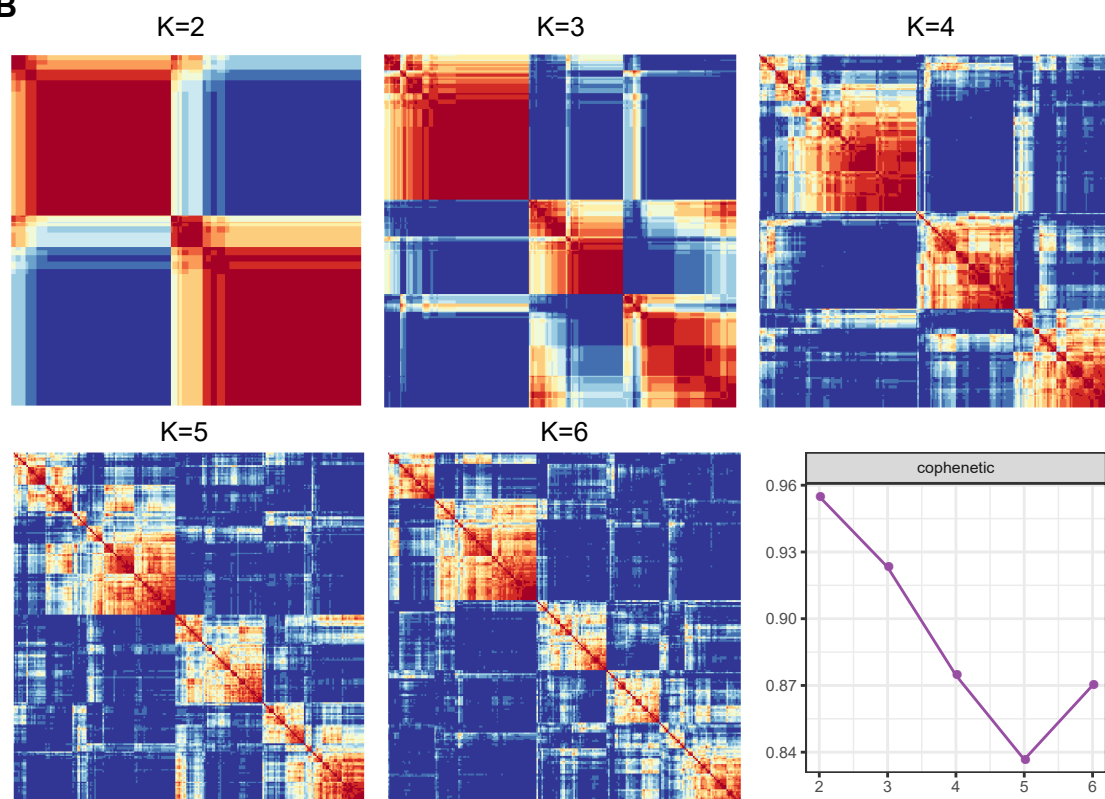

Supplement: Supplementary file 2 — Fig. S2. (A) Consensus matrix of NMF clustering for k = 2–6 in TCGA cohort. (B) Consensus matrix of NMF clustering for k = 2–6 and cophenetic correlation coefficient under corresponding k values in GEO cohort. [file MOL2-14-896-s002.pdf]

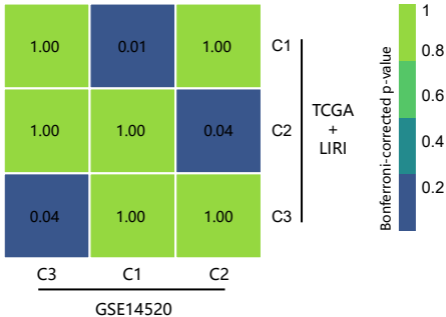

Supplement: Supplementary file 3 — Fig. S3. Submaps matrix shows significant correlation of HCC classification from independent datasets. [file MOL2-14-896-s003.pdf]
