# Supplementary material for: Metabolism‐associated molecular classification of hepatocellular carcinoma
Source: Mol Oncol. 2020 Jan 29;14(4):896–913. doi: 10.1002/1878-0261.12639 (PMC7138397; doi:10.1002/1878-0261.12639)
Supplement: Supplementary file 4 — Fig. S4. Gene Ontology (GO) enrichment analysis of subclass‐specific genes. The x axis indicates the number of genes within each GO term. Detailed information in Table S4. [file MOL2-14-896-s004.pdf]

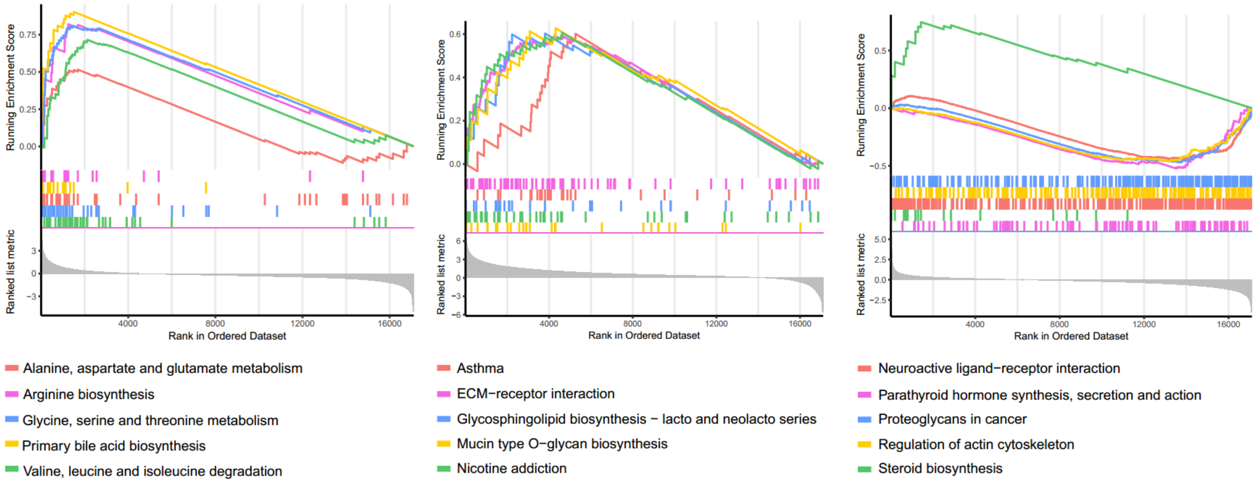
Running Enrichment Score
0.75
0.50
0.25
0.00
Ranked list metric
3
0
−3
4000
8000
12000
16000
Rank in Ordered Dataset
Alanine, aspartate and glutamate metabolism
Arginine biosynthesis
Glycine, serine and threonine metabolism
Primary bile acid biosynthesis
Valine, leucine and isoleucine degradation
Running Enrichment Score
0.6
0.4
0.2
0.0
Ranked list metric
6
3
0
−3
−6
4000
8000
12000
16000
Rank in Ordered Dataset
Asthma
ECM−receptor interaction
Glycosphingolipid biosynthesis − lacto and neolacto series
Mucin type O−glycan biosynthesis
Nicotine addiction
Running Enrichment Score
0.5
0.0
−0.5
Ranked list metric
5.0
2.5
0.0
−2.5
4000
8000
12000
16000
Rank in Ordered Dataset
Neuroactive ligand−receptor interaction
Parathyroid hormone synthesis, secretion and action
Proteoglycans in cancer
Regulation of actin cytoskeleton
Steroid biosynthesis
