# Supplementary material for: Metabolism‐associated molecular classification of hepatocellular carcinoma
Source: Mol Oncol. 2020 Jan 29;14(4):896–913. doi: 10.1002/1878-0261.12639 (PMC7138397; doi:10.1002/1878-0261.12639)
Supplement: Supplementary file 5 — Fig. S5. Results of gene set enrichment analysis of subclass‐specific genes are shown. Detailed information in Table S5. [file MOL2-14-896-s005.pdf]

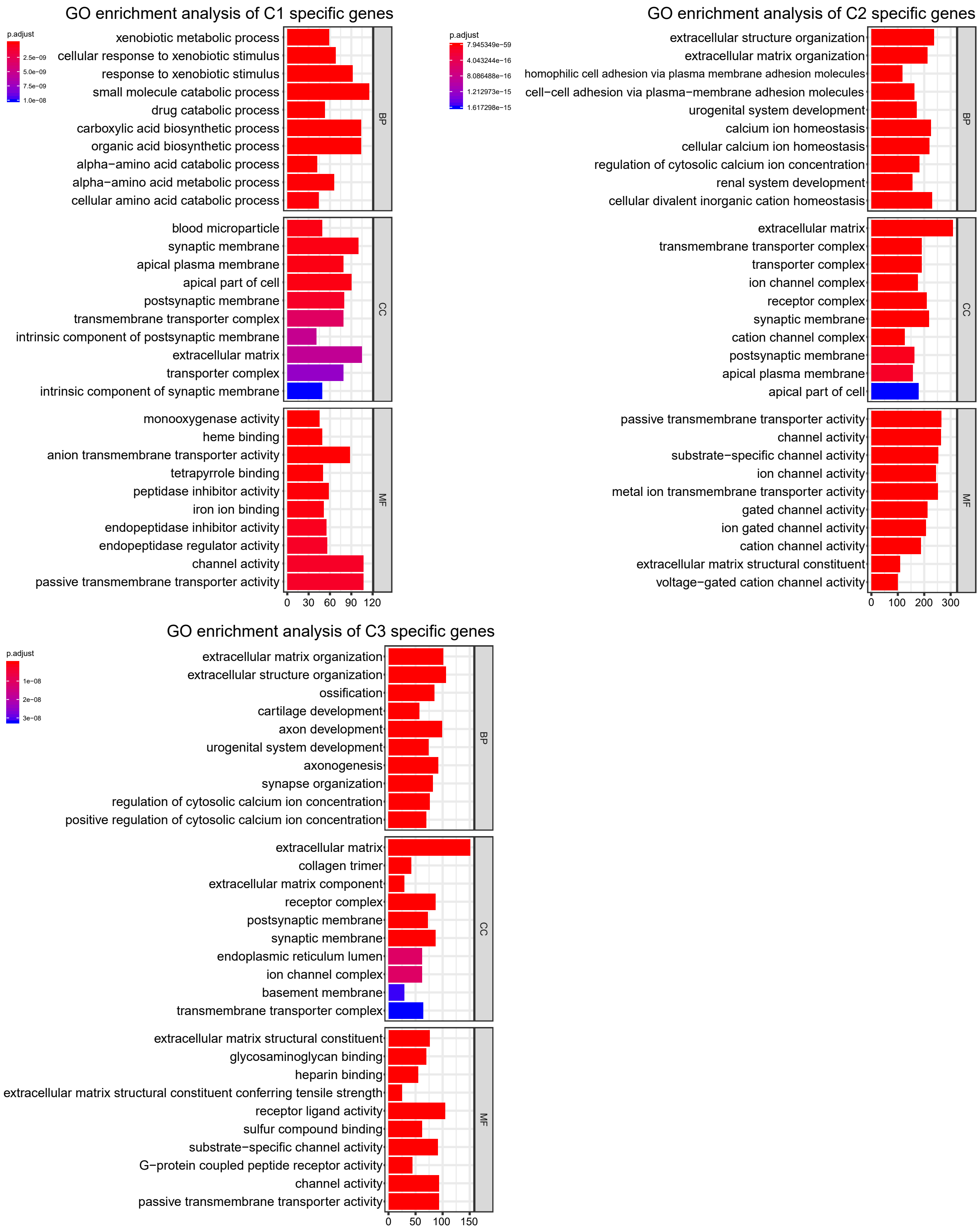
GO enrichment analysis of C1 specific genes
p.adjust
2.5e−09
5.0e−09
7.5e−09
1.0e−08
xenobiotic metabolic process
cellular response to xenobiotic stimulus
response to xenobiotic stimulus
small molecule catabolic process
drug catabolic process
carboxylic acid biosynthetic process
organic acid biosynthetic process
alpha−amino acid catabolic process
alpha−amino acid metabolic process
cellular amino acid catabolic process
BP
blood microparticle
synaptic membrane
apical plasma membrane
apical part of cell
postsynaptic membrane
transmembrane transporter complex
intrinsic component of postsynaptic membrane
extracellular matrix
transporter complex
intrinsic component of synaptic membrane
CC
monooxygenase activity
heme binding
anion transmembrane transporter activity
tetrapyrrole binding
peptidase inhibitor activity
iron ion binding
endopeptidase inhibitor activity
endopeptidase regulator activity
channel activity
passive transmembrane transporter activity
MF
0
30
60
90
120
GO enrichment analysis of C2 specific genes
p.adjust
7.945349e−59
4.043244e−16
8.086488e−16
1.212973e−15
1.617298e−15
extracellular structure organization
extracellular matrix organization
homophilic cell adhesion via plasma membrane adhesion molecules
cell−cell adhesion via plasma−membrane adhesion molecules
urogenital system development
calcium ion homeostasis
cellular calcium ion homeostasis
regulation of cytosolic calcium ion concentration
renal system development
cellular divalent inorganic cation homeostasis
BP
extracellular matrix
transmembrane transporter complex
transporter complex
ion channel complex
receptor complex
synaptic membrane
cation channel complex
postsynaptic membrane
apical plasma membrane
apical part of cell
CC
passive transmembrane transporter activity
channel activity
substrate−specific channel activity
ion channel activity
metal ion transmembrane transporter activity
gated channel activity
ion gated channel activity
cation channel activity
extracellular matrix structural constituent
voltage−gated cation channel activity
MF
0
100
200
300
GO enrichment analysis of C3 specific genes
p.adjust
1e−08
2e−08
3e−08
extracellular matrix organization
extracellular structure organization
ossification
cartilage development
axon development
urogenital system development
axonogenesis
synapse organization
regulation of cytosolic calcium ion concentration
positive regulation of cytosolic calcium ion concentration
BP
extracellular matrix
collagen trimer
extracellular matrix component
receptor complex
postsynaptic membrane
synaptic membrane
endoplasmic reticulum lumen
ion channel complex
basement membrane
transmembrane transporter complex
CC
extracellular matrix structural constituent
glycosaminoglycan binding
heparin binding
extracellular matrix structural constituent conferring tensile strength
receptor ligand activity
sulfur compound binding
substrate−specific channel activity
G−protein coupled peptide receptor activity
channel activity
passive transmembrane transporter activity
MF
0
50
100
150
